# *N*-[2-(Methyl­sulfan­yl)phen­yl]-2-sulfanylbenzamide

**DOI:** 10.1107/S1600536812029765

**Published:** 2012-07-10

**Authors:** Chang-Chih Hsieh, Hon Man Lee, Yih-Chern Horng

**Affiliations:** a National Changhua University of Education, Department of Chemistry, Changhua, Taiwan 50058

## Abstract

In the title compound, C_14_H_13_NOS_2_, the S atom with the methyl group is involved in an intra­molecular hydrogen bond with the amido H atom. In the crystal, the sulfanyl H atoms form inter­molecular hydrogen bonds with the O atoms, connecting the mol­ecules into zigzag chains along the *c* axis. The two aromatic rings exhibit a small interplanar angle of 16.03 (9)°.

## Related literature

For organic and inorganic supramolecules with dynamic covalent bonds: see Huang *et al.* (2012 ▶); Wu *et al.* (2012 ▶). For aromatic amides with N—H⋯S inter­actions: see Du *et al.* (2009 ▶)
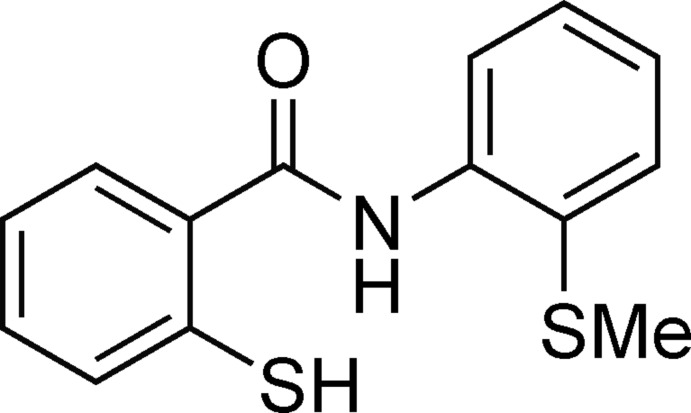


## Experimental

### 

#### Crystal data

C_14_H_13_NOS_2_
*M* *_r_* = 275.37
Monoclinic, 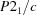

*a* = 7.9549 (5) Å
*b* = 22.7530 (14) Å
*c* = 8.0966 (5) Å
β = 118.787 (1)°
*V* = 1284.36 (14) Å^3^
*Z* = 4
Mo *K*α radiation
μ = 0.40 mm^−1^
*T* = 150 K
0.49 × 0.12 × 0.08 mm

#### Data collection

Bruker SMART APEXII diffractometer
Absorption correction: multi-scan (*SADABS*; Sheldrick, 2003 ▶) *T*_min_ = 0.828, *T*_max_ = 0.969
14799 measured reflections
3196 independent reflections
2577 reflections with *I* > 2σ
*R*_int_ = 0.029

#### Refinement

*R*[*F* ^2^ > 2σ(*F* ^2^)] = 0.038
*wR*(*F* ^2^) = 0.108
*S* = 1.07
3196 reflections
172 parameters
H atoms treated by a mixture of independent and constrained refinement
Δρ_max_ = 0.54 e Å^−3^
Δρ_min_ = −0.24 e Å^−3^

### 

Data collection: *APEX2* (Bruker, 2007 ▶); cell refinement: *SAINT* (Bruker, 2007 ▶); data reduction: *SAINT*; program(s) used to solve structure: *SHELXTL* (Sheldrick, 2008 ▶); program(s) used to refine structure: *SHELXTL*; molecular graphics: *DIAMOND* (Brandenburg, 2006 ▶); software used to prepare material for publication: *SHELXTL*.

## Supplementary Material

Crystal structure: contains datablock(s) I, global. DOI: 10.1107/S1600536812029765/ds2201sup1.cif

Structure factors: contains datablock(s) I. DOI: 10.1107/S1600536812029765/ds2201Isup2.hkl

Supplementary material file. DOI: 10.1107/S1600536812029765/ds2201Isup3.cml

Additional supplementary materials:  crystallographic information; 3D view; checkCIF report

## Figures and Tables

**Table 1:** Hydrogen-bond geometry (Å, °)

*D*—H⋯*A*	*D*—H	H⋯*A*	*D*⋯*A*	*D*—H⋯*A*
N1—H8⋯S1	0.83 (2)	2.49 (2)	2.9150 (14)	112.7 (17)
S2—H13⋯O1^i^	1.24 (3)	2.37 (3)	3.5976 (14)	169.5 (17)

Symmetry code: (i) 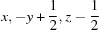.

**Fig. 1.**
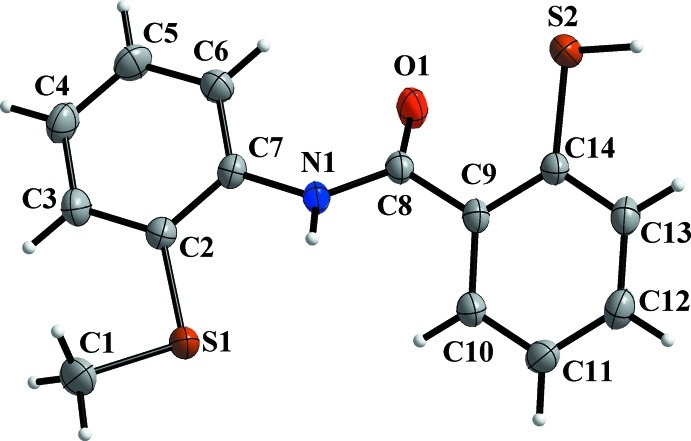
The structure of the title compound, showing 50% probability displacement ellipsoids for the non-hydrogen atoms. The H atoms are dipicted by circles of an arbitrary radius.

**Fig. 2.**
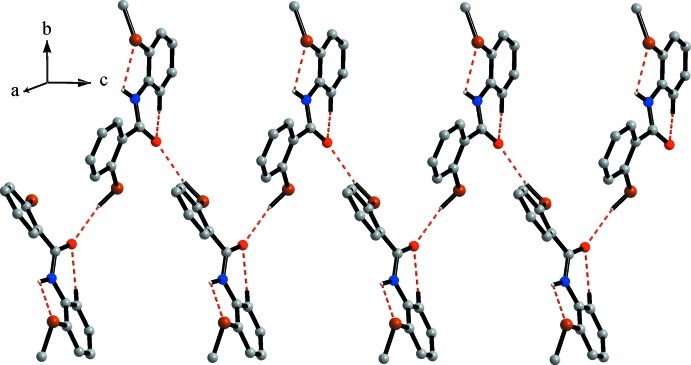
A view of the one-dimensional zigzag hydrogen-bonded chain, displaying the hydrogen bonds as dashed lines.

## supplementary crystallographic information

### Comment

The N—H···S hydrogen bonding interactions are quite often found in proteins, where the sulfur atoms are ususally from cysteine or methionine residues. Many organic compounds containing amide and thiol (or thioether) moieties were investigated to give a deep insight of the N—H···S hydrogen bonding interactions (Du *et al.* 2009), which may help to understand protein folding processes and enzymatic catalyses. Our group is interesting in the preparation and encapsulation behaviors of organic and inorganic supramolecules with dynamic covalent bonds (Huang *et al.* 2012; Wu *et al.* 2012). The title compound is a sulfur-containing secondary amide with two aryl groups. We synthesized and report its structure here, and will atempt to use it as a building block for the construction of more complex organic or inorganic molecules with unique properties.

The title compound crystallizes in the monoclinic space group *P* 2_1_/*c*. Fig. 1. shows a displacement ellipsoid plot of the compound. The S—C(*sp*^3^) bond distane of 1.7876 (19) Å is slightly longer than those of the S—C(*sp*^2^) bonds [1.7678 (17) and 1.7708 (17) Å]. The two aromatic rings (C2 to C7) and (C9 to C14) exhibit a small interplanar angle of 16.03 (9) °. Both classical and non-classical hydrogen bonds are present (Table 1). The S atom with the Me group involves in an intramolecular hydrogen bond with the amido H-atom [N···S = 2.9150 (14) Å]. A non-classical intramolecular hydrogen bond of the type C—H···O also exists [C···O = 2.949 (2) Å]. In the crystal structure, the H atoms upon S atoms form intermolecular hydrogen bonds with the O atoms [S···O = 3.5976 (14) Å], connecting the compounds into zigzag chains along the *c* axis (Fig. 2).

### Experimental

A CH_2_Cl_2_ solution (20 ml) containing 2-methylthioaniline (1.39 g, 10 mmol) and NEt_3_ (1.02 g, 10 mmol) was mixed with another CH_2_Cl_2_ solution (20 ml) containing 2,2'-dithiosalicyl chloride (1.7 g, 5 mmol). After stirred at room temperature for 12 h, the mixture was washed with saturated NaHCO_3_ solution and distilled water. The combined CH_2_Cl_2_ portions were collected and dried with anhydrous MgSO_4_. The solvent was then removed under vacuum to afford a yellow powder. An uncapped 50 ml flask, containing the yellow solid and an excess NaBH_4_ (0.33 g, 9 mmol), was placed in an ice-water bath. To this flask, 25 ml of MeOH was added slowly. After the resulting mixture stirred at 4°C for 10 minutes, the water bath was removed and the stirring was continued for another 30 minutes. The yellowish mixture was added dropwise with concentrated HCl_(_*_aq_*_)_ to quench excess NaBH_4_. After completion, the solution was extracted with CH_2_Cl_2_ and distilled water. The collected CH_2_Cl_2_ fractions were dried over anhydrous MgSO_4_, filtered, and vacuum dried to give 2.03 g of light-yellow solid (86% yield). Crystals suitable for X-ray diffraction analysis were obtained by slow evaporation of THF solution of the compound at -4°C.

### Refinement

The H on C atoms were positioned geometrically and refined as riding atoms, with C_aryl_—H = 0.93 and C_methyl_—H = 0.96 Å while *U*_iso_(H) = 1.2*U*_eq_ (C_aryl_) and 1.5*U*_eq_ (C_methyl_). The H on N and S atoms were located from the difference Fourier map and freely refined (N1—H8 = 0.83 (2) Å and S2—H13 = 1.24 (3) Å).

### Crystal data

**Table:**

C_14_H_13_NOS_2_	*F*(000) = 576
*M**_r_* = 275.37	*D*_x_ = 1.424 Mg m^−^^3^
Monoclinic, *P*2_1_/*c*	Mo *K*α radiation, λ = 0.71073 Å
Hall symbol: -P 2ybc	Cell parameters from 3996 reflections
*a* = 7.9549 (5) Å	θ = 2.9–28.7°
*b* = 22.7530 (14) Å	µ = 0.40 mm^−^^1^
*c* = 8.0966 (5) Å	*T* = 150 K
β = 118.787 (1)°	Block, light-yellow
*V* = 1284.36 (14) Å^3^	0.49 × 0.12 × 0.08 mm
*Z* = 4	

### Data collection

**Table:**

Bruker SMART APEXII diffractometer	3196 independent reflections
Radiation source: fine-focus sealed tube	2577 reflections with *I* > 2σ
Graphite monochromator	*R*_int_ = 0.029
ω scans	θ_max_ = 28.3°, θ_min_ = 1.8°
Absorption correction: multi-scan (*SADABS*; Sheldrick, 2003)	*h* = −10→10
*T*_min_ = 0.828, *T*_max_ = 0.969	*k* = −30→29
14799 measured reflections	*l* = −10→10

### Refinement

**Table:**

Refinement on *F*^2^	Primary atom site location: structure-invariant direct methods
Least-squares matrix: full	Secondary atom site location: difference Fourier map
*R*[*F*^2^ > 2σ(*F*^2^)] = 0.038	Hydrogen site location: inferred from neighbouring sites
*wR*(*F*^2^) = 0.108	H atoms treated by a mixture of independent and constrained refinement
*S* = 1.07	*w* = 1/[σ^2^(*F*_o_^2^) + (0.0645*P*)^2^ + 0.2868*P*] where *P* = (*F*_o_^2^ + 2*F*_c_^2^)/3
3196 reflections	(Δ/σ)_max_ = 0.011
172 parameters	Δρ_max_ = 0.54 e Å^−^^3^
0 restraints	Δρ_min_ = −0.24 e Å^−^^3^

### Special details

**Table:**

Geometry. All e.s.d.'s (except the e.s.d. in the dihedral angle between two l.s. planes) are estimated using the full covariance matrix. The cell e.s.d.'s are taken into account individually in the estimation of e.s.d.'s in distances, angles and torsion angles; correlations between e.s.d.'s in cell parameters are only used when they are defined by crystal symmetry. An approximate (isotropic) treatment of cell e.s.d.'s is used for estimating e.s.d.'s involving l.s. planes.
Refinement. Refinement of *F*^2^ against ALL reflections. The weighted *R*-factor *wR* and goodness of fit *S* are based on *F*^2^, conventional *R*-factors *R* are based on *F*, with *F* set to zero for negative *F*^2^. The threshold expression of *F*^2^ > σ(*F*^2^) is used only for calculating *R*-factors(gt) *etc*. and is not relevant to the choice of reflections for refinement. *R*-factors based on *F*^2^ are statistically about twice as large as those based on *F*, and *R*- factors based on ALL data will be even larger.

### Fractional atomic coordinates and isotropic or equivalent isotropic displacement parameters (Å^2^)

**Table:**

	*x*	*y*	*z*	*U*_iso_*/*U*_eq_	
S1	0.60030 (6)	−0.051148 (18)	0.90758 (6)	0.02864 (13)	
S2	0.98708 (6)	0.236365 (19)	0.99798 (7)	0.03244 (14)	
C9	0.6995 (2)	0.15183 (7)	0.8772 (2)	0.0221 (3)	
C14	0.7592 (2)	0.20651 (7)	0.8423 (2)	0.0238 (3)	
C13	0.6343 (3)	0.23749 (7)	0.6799 (2)	0.0280 (4)	
H12	0.6733	0.2732	0.6538	0.034*	
C7	0.9140 (2)	0.01217 (7)	1.1500 (2)	0.0231 (3)	
C10	0.5160 (2)	0.13120 (7)	0.7532 (2)	0.0260 (3)	
H9	0.4759	0.0953	0.7769	0.031*	
C8	0.8306 (2)	0.11675 (7)	1.0481 (2)	0.0229 (3)	
C2	0.8275 (2)	−0.04347 (7)	1.1096 (2)	0.0243 (3)	
C11	0.3925 (2)	0.16322 (8)	0.5952 (3)	0.0311 (4)	
H10	0.2695	0.1494	0.5152	0.037*	
C3	0.9226 (3)	−0.09044 (8)	1.2301 (3)	0.0308 (4)	
H4	0.8674	−0.1276	1.2032	0.037*	
C4	1.0979 (3)	−0.08202 (8)	1.3888 (3)	0.0353 (4)	
H5	1.1598	−0.1134	1.4688	0.042*	
C6	1.0906 (2)	0.02020 (8)	1.3094 (2)	0.0294 (4)	
H7	1.1480	0.0571	1.3361	0.035*	
C12	0.4545 (3)	0.21603 (8)	0.5579 (3)	0.0315 (4)	
H11	0.3743	0.2371	0.4498	0.038*	
C1	0.6034 (3)	−0.12570 (8)	0.8394 (3)	0.0417 (5)	
H3	0.7172	−0.1325	0.8295	0.063*	
H1	0.4924	−0.1330	0.7198	0.063*	
H2	0.6021	−0.1516	0.9324	0.063*	
C5	1.1817 (3)	−0.02660 (9)	1.4290 (3)	0.0355 (4)	
H6	1.2993	−0.0209	1.5364	0.043*	
N1	0.8202 (2)	0.05779 (6)	1.0186 (2)	0.0234 (3)	
O1	0.9345 (2)	0.14000 (5)	1.19986 (18)	0.0374 (3)	
H8	0.749 (3)	0.0455 (9)	0.910 (3)	0.035 (6)*	
H13	0.969 (4)	0.2748 (11)	0.881 (4)	0.058 (7)*	

### Atomic displacement parameters (Å^2^)

**Table:**

	*U*^11^	*U*^22^	*U*^33^	*U*^12^	*U*^13^	*U*^23^
S1	0.0252 (2)	0.0206 (2)	0.0330 (2)	0.00099 (15)	0.00836 (18)	−0.00087 (16)
S2	0.0276 (2)	0.0233 (2)	0.0391 (3)	−0.00423 (16)	0.01020 (19)	0.00233 (17)
C9	0.0257 (8)	0.0174 (7)	0.0250 (8)	0.0031 (6)	0.0135 (7)	0.0010 (6)
C14	0.0238 (7)	0.0185 (8)	0.0289 (8)	0.0014 (6)	0.0124 (7)	−0.0001 (6)
C13	0.0325 (9)	0.0212 (8)	0.0328 (9)	0.0051 (6)	0.0178 (8)	0.0057 (7)
C7	0.0254 (8)	0.0208 (8)	0.0237 (8)	0.0040 (6)	0.0123 (6)	0.0023 (6)
C10	0.0274 (8)	0.0194 (7)	0.0307 (9)	0.0003 (6)	0.0136 (7)	−0.0003 (6)
C8	0.0246 (8)	0.0183 (7)	0.0271 (8)	0.0015 (6)	0.0134 (7)	0.0009 (6)
C2	0.0236 (8)	0.0224 (8)	0.0261 (8)	0.0013 (6)	0.0115 (7)	0.0009 (6)
C11	0.0254 (8)	0.0282 (9)	0.0321 (9)	0.0028 (7)	0.0078 (7)	−0.0024 (7)
C3	0.0328 (9)	0.0237 (8)	0.0348 (9)	0.0012 (7)	0.0154 (8)	0.0057 (7)
C4	0.0363 (10)	0.0322 (10)	0.0315 (10)	0.0089 (7)	0.0117 (8)	0.0101 (7)
C6	0.0294 (9)	0.0254 (8)	0.0289 (9)	−0.0015 (7)	0.0105 (7)	−0.0030 (7)
C12	0.0342 (9)	0.0270 (8)	0.0292 (9)	0.0093 (7)	0.0119 (8)	0.0058 (7)
C1	0.0379 (11)	0.0281 (9)	0.0467 (12)	−0.0012 (8)	0.0106 (9)	−0.0110 (8)
C5	0.0318 (9)	0.0380 (10)	0.0261 (9)	0.0036 (8)	0.0053 (7)	0.0045 (7)
N1	0.0262 (7)	0.0169 (6)	0.0231 (7)	0.0006 (5)	0.0087 (6)	0.0005 (5)
O1	0.0450 (8)	0.0210 (6)	0.0295 (7)	0.0010 (5)	0.0046 (6)	−0.0030 (5)

### Geometric parameters (Å, º)

**Table:**

S1—C2	1.7678 (17)	C8—N1	1.358 (2)
S1—C1	1.7876 (19)	C2—C3	1.399 (2)
S2—C14	1.7708 (17)	C11—C12	1.386 (3)
S2—H13	1.24 (3)	C11—H10	0.9300
C9—C10	1.395 (2)	C3—C4	1.381 (3)
C9—C14	1.408 (2)	C3—H4	0.9300
C9—C8	1.500 (2)	C4—C5	1.390 (3)
C14—C13	1.398 (2)	C4—H5	0.9300
C13—C12	1.378 (3)	C6—C5	1.386 (3)
C13—H12	0.9300	C6—H7	0.9300
C7—C6	1.388 (2)	C12—H11	0.9300
C7—C2	1.402 (2)	C1—H3	0.9600
C7—N1	1.415 (2)	C1—H1	0.9600
C10—C11	1.386 (2)	C1—H2	0.9600
C10—H9	0.9300	C5—H6	0.9300
C8—O1	1.222 (2)	N1—H8	0.83 (2)
			
C2—S1—C1	102.68 (9)	C12—C11—H10	120.4
C14—S2—H13	91.3 (12)	C4—C3—C2	120.52 (17)
C10—C9—C14	119.32 (15)	C4—C3—H4	119.7
C10—C9—C8	120.43 (14)	C2—C3—H4	119.7
C14—C9—C8	120.24 (14)	C3—C4—C5	119.96 (17)
C13—C14—C9	118.60 (15)	C3—C4—H5	120.0
C13—C14—S2	119.75 (13)	C5—C4—H5	120.0
C9—C14—S2	121.64 (12)	C5—C6—C7	120.20 (17)
C12—C13—C14	121.19 (16)	C5—C6—H7	119.9
C12—C13—H12	119.4	C7—C6—H7	119.9
C14—C13—H12	119.4	C13—C12—C11	120.40 (16)
C6—C7—C2	119.94 (15)	C13—C12—H11	119.8
C6—C7—N1	122.27 (15)	C11—C12—H11	119.8
C2—C7—N1	117.70 (14)	S1—C1—H3	109.5
C11—C10—C9	121.21 (16)	S1—C1—H1	109.5
C11—C10—H9	119.4	H3—C1—H1	109.5
C9—C10—H9	119.4	S1—C1—H2	109.5
O1—C8—N1	123.96 (15)	H3—C1—H2	109.5
O1—C8—C9	122.02 (14)	H1—C1—H2	109.5
N1—C8—C9	114.01 (14)	C6—C5—C4	120.21 (17)
C3—C2—C7	119.15 (16)	C6—C5—H6	119.9
C3—C2—S1	122.59 (13)	C4—C5—H6	119.9
C7—C2—S1	118.26 (12)	C8—N1—C7	128.83 (14)
C10—C11—C12	119.22 (16)	C8—N1—H8	118.1 (15)
C10—C11—H10	120.4	C7—N1—H8	113.1 (15)
			
C10—C9—C14—C13	−2.2 (2)	C1—S1—C2—C3	−30.12 (18)
C8—C9—C14—C13	178.94 (15)	C1—S1—C2—C7	150.33 (15)
C10—C9—C14—S2	177.94 (13)	C9—C10—C11—C12	1.5 (3)
C8—C9—C14—S2	−0.9 (2)	C7—C2—C3—C4	1.1 (3)
C9—C14—C13—C12	1.5 (3)	S1—C2—C3—C4	−178.41 (14)
S2—C14—C13—C12	−178.68 (14)	C2—C3—C4—C5	−0.5 (3)
C14—C9—C10—C11	0.8 (3)	C2—C7—C6—C5	−0.1 (3)
C8—C9—C10—C11	179.58 (15)	N1—C7—C6—C5	−176.62 (16)
C10—C9—C8—O1	−140.86 (17)	C14—C13—C12—C11	0.8 (3)
C14—C9—C8—O1	37.9 (2)	C10—C11—C12—C13	−2.3 (3)
C10—C9—C8—N1	38.2 (2)	C7—C6—C5—C4	0.8 (3)
C14—C9—C8—N1	−143.00 (15)	C3—C4—C5—C6	−0.5 (3)
C6—C7—C2—C3	−0.9 (3)	O1—C8—N1—C7	3.2 (3)
N1—C7—C2—C3	175.84 (15)	C9—C8—N1—C7	−175.82 (15)
C6—C7—C2—S1	178.70 (13)	C6—C7—N1—C8	−28.8 (3)
N1—C7—C2—S1	−4.6 (2)	C2—C7—N1—C8	154.54 (17)

### Hydrogen-bond geometry (Å, º)

**Table:**

*D*—H···*A*	*D*—H	H···*A*	*D*···*A*	*D*—H···*A*
N1—H8···S1	0.83 (2)	2.49 (2)	2.9150 (14)	112.7 (17)
S2—H13···O1^i^	1.24 (3)	2.37 (3)	3.5976 (14)	169.5 (17)
C6—H7···O1	0.93	2.42	2.949 (2)	116

Symmetry code: (i) *x*, −*y*+1/2, *z*−1/2.

## References

[bb1] Brandenburg, K. (2006). *DIAMOND* Crystal Impact GbR, Bonn, Germany.

[bb2] Bruker (2007). *APEX2* and *SAINT* Bruker AXS Inc., Madison, Wisconsin, USA.

[bb3] Du, P., Jiang, X.-K. & Li, Z.-T. (2009). *Tetrahedron Lett.* **50**, 320–324.

[bb4] Huang, P.-S., Kuo, C.-H., Hsieh, C.-C. & Horng, Y.-C. (2012). *Chem. Commun.* **48**, 3227–3229. 10.1039/c2cc17716a 22331261

[bb5] Sheldrick, G. M. (2003). *SADABS* University of Göttingen, Germany.

[bb6] Sheldrick, G. M. (2008). *Acta Cryst.* A**64**, 112–122. 10.1107/S0108767307043930 18156677

[bb7] Wu, Z.-S., Hsu, J.-T., Hsieh, C.-C. & Horng, Y.-C. (2012). *Chem. Commun.* **48**, 3436–3438. 10.1039/c2cc30381g 22358389

